# Mapping the EORTC item library to the World Health Organization international classification of functioning, disability and health: a content analysis

**DOI:** 10.1007/s11136-026-04335-4

**Published:** 2026-07-23

**Authors:** Hayat Hamzeh, Shaista Meer, Maria Rothmund-Grenier, Nathalie Egeter, Emma Lidington, Dagmara Kuliś, Mogens Groenvold, Johannes M. Giesinger, Alexandra Gilbert, Claire Piccinin

**Affiliations:** 1 Leeds Institute for Medical Research, University of Leeds, St James’s University Hospital, Level 5, Clinical Sciences Building, Beckett Street, Leeds, LS9 7TF UK; 2 University Hospital of Psychiatry II, Medical University of Innsbruck, Anichstraße 35, 6020 Innsbruck, Austria; 3 Cancer Prevention Trials Unit, Queen Mary University of London, Empire House, 67-75 New Rd, London, E1 1HH UK; 4 Quality of Life Department, European Organisation for Research and Treatment of Cancer (EORTC) Headquarters, Brussels, Belgium; 5 Department of Public Health, University of Copenhagen, Copenhagen, Denmark; 6 Palliative Care Research Unit, Bispebjerg and Frederiksberg Hospital, Copenhagen, Denmark

**Keywords:** Health-related quality of life, Patient-reported outcomes, PROM, Cancer, Oncology, ICF

## Abstract

**Purpose:**

The European Organisation for Research and Treatment of Cancer (EORTC) questionnaires are some of the most widely used patient-reported outcome measures (PROMs) for health-related quality of life assessment in oncology. The EORTC Item Library is an online platform comprising all EORTC PROMs that enables the creation of customised questionnaires (item lists). To characterise and better understand the breadth of functioning, disability and health coverage within the EORTC Item Library, this study aimed to link and analyse its content using the International Classification of Functioning, Disability and Health (ICF).

**Methods:**

A team of reviewers applied the most recent ICF linking rules to map the items currently included in the EORTC Item Library. Descriptive analysis was used to summarise the content covered in ICF categories and concepts coded as not covered or not definable.

**Results:**

The 1076 EORTC items covered 1860 concepts overall, with most (n = 1641, 88.2%) linked to the ICF. Concepts were linked to the majority of ICF chapters (n = 28/30, 93.3%), covering 60.9% (168/276) of all second-level ICF categories. The components of ‘b-Body functions’ and ‘d-Activities and participation’ had the highest coverage, with concepts linked to 80.0% (64/80) and 68.5% (63/92) of all second-level categories respectively.

**Conclusions:**

The EORTC Item Library provides broad coverage of functioning, disability and health within the ICF. Using the ICF to describe its content can inform the selection of EORTC items and PROMs and facilitate standardised comparison across measures and contexts, supporting the use and interpretation of EORTC measures in research and clinical practice.

## Introduction

Patient reported outcome measures (PROMs) play a key role in assessing the impact of cancer and its therapies from a patient-centred perspective. Current regulatory guidelines emphasise the use of PROMs to provide a more accurate assessment in oncology research and clinical care, complementing other clinical outcomes [1, 2]. This is reflected by their increased implementation within oncology research settings, including clinical trials and observational studies, in addition to clinical care [3–6]. A wide range of PROMs have been developed using rigorous methods to assess health-related quality of life (HRQoL) in oncology across diverse populations [7]. These capture general cancer issues, disease-specific, and treatment-related symptoms and adverse events, in addition to impacts on role, physical and other types of functioning important to patients’ lives [8].

The European Organisation for Research and Treatment of Cancer (EORTC) Item Library is an interactive online platform developed by the EORTC Quality of Life Group to support HRQoL measurement in oncology [9]. In addition to displaying the portfolio of available PROMs, it supports the creation of customised questionnaires (i.e., item lists) from the pool of available items. The EORTC Item Library has become a critical tool in oncology clinical trials, particularly for capturing treatment-specific side effects and patient experiences not covered by existing validated questionnaires [10]. Customised item lists are increasingly used alongside the EORTC core questionnaire (QLQ-C30) and disease-specific modules to assess additional concepts relevant to the research questions and context of use. EORTC item lists have been used to enhance conceptual coverage of various health areas, thus supporting population-specific content validity and improving sensitivity to treatment effects, particularly in trials involving novel treatments and rare disease populations [11].

Beyond research, Item Library-derived item lists are also used in routine cancer care to monitor patient well-being and guide treatment decisions [12]. Their integration into electronic patient-reported outcome (PRO) platforms enables real-time symptom tracking and enhances communication between patients and providers [13]. Clinicians who are registered Item Library users can select items relevant to individual patients to create customised item lists to use for monitoring adverse events and tailoring supportive care interventions.

Since the EORTC Item Library covers aspects of functioning, disability and health, in addition to general HRQoL, evaluating its conceptual breadth and ensuring comparability with other PROMs and health outcomes measures requires alignment with internationally recognised frameworks. The World Health Organization (WHO) has developed two complementary classification systems to support communication and standardisation across healthcare systems. One is the International Classification of Diseases (ICD) which is used to classify diseases, disorders, injuries, and causes of death [14]. The other is the International Classification of Functioning, Disability and Health (ICF), which offers a universal biopsychosocial framework for describing functioning, disability and health across populations and settings [15, 16]. The ICF encompasses body functions, body structures, activities and participation, and environmental factors, promoting standardised communication and comparability in health measurement [17]. It enables a universal language for health professionals, researchers, and policymakers, facilitating communication, data comparison, and policy development [18]. To ensure transparency and consistency in mapping PROMs to ICF categories, structured linking rules have been introduced to analyse and compare the content of healthcare measurement tools [19–21].

Multiple measures from the EORTC Item Library, including the EORTC CAT Core and QLQ-C30, have been linked to the ICF in previous work, with these assessments focused on coverage of ICF chapters and categories related to physical, role, social and emotional functioning [22–24] and fatigue [25]. Indeed, by providing a common conceptual framework for EORTC PROMs and other PROMs outside of the EORTC portfolio, these efforts have supported subsequent work which linked physical functioning scores from frequently used PROMs in cancer settings [26]. The results of this work can be used to compare physical functioning scores across PROMs and pool data, and future work may aim to establish additional crosswalks for PROM scores summarising other HRQoL areas. Furthermore, prior research mapping the EORTC Item Library to the Common Terminology Criteria for Adverse Events (CTCAE), identified over 300 (out of 950) items assigned a non-CTCAE classification [27]. With its focus on adverse events, the CTCAE is limited in scope, highlighting the need to map to additional classification systems to improve alignment across widely used frameworks.

In this study, we systematically linked all items currently included in the EORTC Item Library to the ICF using the most recent ICF linking rules [19]. The primary aim was to examine the extent to which the EORTC Item Library captures the biopsychosocial perspectives related to functioning disability, and health, including contextual factors, using the ICF as a benchmark for conceptual coverage. The work was designed to complement existing mapping frameworks and broaden the interpretability and applicability of the EORTC Item Library.

## Methods

### Description of the EORTC item library items

The EORTC Item Library is an online platform currently comprising over 1000 validated items (questions) derived from more than 70 validated questionnaires developed to measure different aspects of HRQoL in diverse cancer populations [28, 29]. These items are designed to assess HRQoL and its related components, like symptom burden and satisfaction with care, in individuals with cancer across various cancer types and treatment modalities. A standard EORTC item is a single PRO question designed according to the EORTC Quality of Life Group’s formal guidelines [30]. A standard item is available as an English source version, and multiple linguistically validated translations are accessible directly through the Item Library. To facilitate creating item lists and questionnaires for tailored use, EORTC items follow a generally similar format. A standard EORTC item has:

1. A clearly defined question (item wording):

Each item consists of a standardized question generally assessing a specific symptom, functional status, or quality of life issue; for example: Have you had pain?

2. Standard response scale:

Most EORTC items use the 4‑point Likert response scale: Not at all / A little / Quite a bit / Very much.

3. Standard time frame:

Most EORTC items assess experiences “during the past week” (or another predefined time scale when appropriate).

### Description of the ICF

The ICF provides a standardised way to document and communicate information about functioning, disability and health. It has a hierarchical structure that is divided into the following components:

1. Body functions (b codes) refer to the physiological functions of body systems, including psychological functions.
2. Body structures (s codes) refer to anatomical parts of the body such as organs, limbs, and their parts.
3. Activities and participation (d codes) describe a person’s ability to perform tasks and engage in life situations.
4. Environmental factors (e codes) refer to the physical, social, and attitudinal environment that affects functioning.

**Fig. 1:**
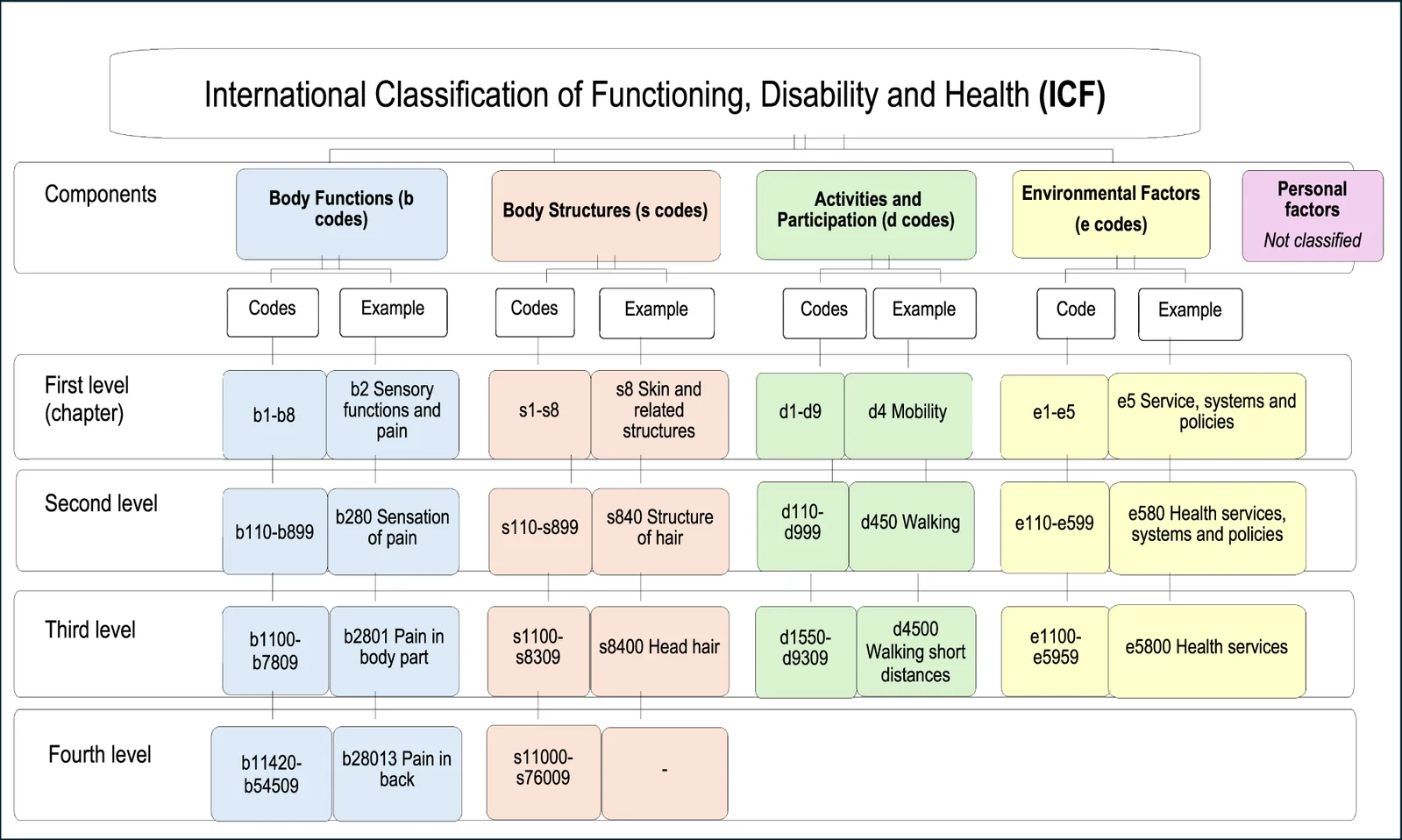
The components, hierarchy and coding system of the ICF

The ICF has a hierarchical structure (Fig. 1) wherein each of the components consists of multiple chapters. Each chapter is divided into categories which can be branched to three levels, or four levels in some cases, to allow for increasing specificity when describing aspects of functioning, disability, and health. Chapters in the ICF components ‘b-Body functions’ and ‘d‐Activities and participation’ are organised in blocks (domains) in which the categories are clustered in an ordered way. Each ICF category is given an alphanumerical code that begins with a lowercase letter (b, s, d, e) according to the corresponding component, in addition to numbers reflecting its category and level. For example, b7651 is the code for the category of ‘Tremor’.

In addition to the components, the ICF includes ‘personal factors’, which describe the background characteristics that may affect a person’s functioning and experience of health, like education and coping styles. Unlike the ICF components, ‘personal factors’ are not classified into different categories and levels.

### Linking the EORTC item library content to the ICF

The linking process was applied to the content of the EORTC Item Library in December 2025. Some of the EORTC Item Library content was previously linked to the ICF [22–25], leaving a total of 819 items not linked. All data from previous linking work were imported and used in this analysis, in addition to the 819 items which were coded by a team of four reviewers (HH, SM, EL, CP).

We applied the same linking methods used in the previous ICF-linking publications [22–25], using the most recent ICF linking rules [19] (see Table 1). This process included identifying the main concepts and additional meaningful concepts of each item and then linking the meaningful concepts to the most precise ICF categories. If the most precise category for a meaningful concept was an ICF level three or four category, the corresponding ICF component, level one, and level two codes were also recorded. Based on the ICF classification, items assigned to ‘personal factors’ were not further classified and the main attributes contained in each item were recorded, for example, religious beliefs. If a concept was clearly not included in the ICF categories, it was classified as ‘not covered’. When applicable, those items were further categorised based on their main domain, for example, global quality of life and health condition. When the concept was not specific enough for the reviewers to decide on the most suitable ICF category, it was classified as ‘not definable’. When applicable, these concepts were further categorised according to their main domain, for example, general side effects.

**Table 1:** Application of the most recent ICF linking rules by Cieza and colleagues 2019 [19]: examples from the linking exercise performed by the reviewers

Rule	Application of the linking rule in this study	Example	
		EORTC Item Library item	ICF code(s)
1. Acquire good knowledge of the fundamentals of the ICF, chapters, domains and categories of the detailed classification, before starting to link meaningful concepts to the ICF categories	All reviewers have previous experience in using the ICF in clinical or academic settings, and/or previous experience in ICF linking projects. The following linking rules were applied to each item by at least two reviewers		
2. Identify the main concept(s) most relevant to be linked to the ICF	The reviewers identified the main concept for each item based on the rule: by answering the question: What is this item about?	Has your voice sounded different as a result of your disease or treatment? (Q944)	Main concept: Quality of voice ICF category: b3101 Quality of voice
3. Identify any additional concepts to the main concept(s) already identified in the previous step	Additional concepts were identified for each item	Did you have difficulty reading because of your vision? (Q221)	Main concept: vision ICF category: b210 Seeing functions Additional concept: Reading ICF category: d116 Reading
4. Identify and document the perspective taken on within a certain piece of information when linking it to the ICF	Due to the large number of items, analysis of perspectives was not performed for all items Perspectives could be classified using different categories, i.e.: Appraisal: the extent to which a person’s expectations have been satisfied	Have you felt satisfied with the quality of your social life (including family and/ or friends)? (Q388)	Appraisal
	Need or dependency: the needs an individual requires because of their condition	Did you need help with household chores such as cleaning or shopping? (Q37)	Need or dependency
	Descriptive (Performance): describing what an individual does in his/her given environment	Have you had difficulty using a computer /laptop /tablet? (Q1054)	Descriptive perspective: Performance
	Descriptive (Capacity): Item describes an individual’s ability to execute a specific task or an action	Do you have any trouble walking 100 m? (Q627)	Descriptive perspective: Capacity
5. Identify and document the categorization of the response options	The EORTC items generally use the same response scale. Due to the large number of items, response scale analysis for all items was not performed	The response scale most frequently used in EORTC items is: not at all, a little, quite a bit, very much	The response scale could represent intensity or frequency of the problem
6. Link all meaningful concepts, the most relevant and additional ones, to the most precise ICF category	The ICF online browser [39] was used to search for a category for each meaningful concept. If available, the concept was linked to the most precise ICF category	Have you been in too much pain to eat? (Q302)	Main concept: pain ICF category: b280 Sensation of pain Additional concept: Eating ICF category: d550 Eating
7. Use ‘‘other specified [8]’’ or ‘‘unspecified [9]’’ ICF categories as appropriate	If the concept is contained in ICF category but is more specific, it was linked to “other specified” If the concept is related to an ICF category but more details are needed for specific coding, it was linked to “not specified”	Have you had aches or pains in your muscles or bones? (Q528)	b28018 Pain in body part, other specified (pain muscles or bones)
		Have you become mentally stronger? (Q1075)	b199 Mental functions, unspecified (mental strength)
9. If the information provided by the meaningful concept is not sufficient for making a decision about the most precise ICF category, assign the concept to nd (not definable)	Concepts were categorised as not definable (nd) per the rule. Then, when applicable, they were further classified according to their domain into: General Health (nd-gh) Physical health (nd-ph) Mental health (nd-mh)	Have you had side-effects from your treatment? (Q525)	Main concept: side effects Most precise ICF category: nd
		Were you worried about your health in the future? (Q41)	Main concept: general health Most precise ICF category: nd-gh
11. If the meaningful concept is not contained in the ICF, but is clearly a personal factor as defined in the ICF, assign the meaningful concept to pf (personal factors)	Concepts were categorised as personal factors if they included attributes that could affect attitudes toward treatment, like religious and spiritual beliefs and motivation for treatment	I believe in God or in someone or something greater than myself (Q615) Have you felt motivated to live life to the full? (Q1077)	Main concept: Belief in God Most precise ICF category: pf Main concept: motivation Most precise ICF category: pf
12. If the meaningful concept is not contained in the ICF, assign this meaningful concept to nc (not covered)	Concepts not included in the ICF were assigned to not covered (nc), and when applicable, they were classified into: Not covered- quality of life (nc-qol) Not covered-health condition (nc-hc)	How much has your disease been a burden to you? (Q46)	Main concept: disease burden Most precise ICF category: nc-hc Not covered-health condition
		How would you rate your overall quality of life during the past week? (Q33)	Main concept: Quality of life Most precise ICF category: nc-qol Not covered-quality of life

Each item was coded by two reviewers, and any disagreements were resolved by a third. Two reviewers (HH, SM) combined and synthesised the codes for all items from previous work and publications and carried out coding of new items that had not yet been linked to the ICF. Later, they performed a final review for consistency and alignment with the linking rules. Any disagreements between the two reviewers were resolved by discussion with a third reviewer. Interrater agreement was calculated using percentage agreement wherein the number of agreements was divided by the number of assessments and multiplied by 100. Agreement was based on the two reviewers who performed the final review and calculated for level one, level two, and level three ICF categories.

As many items contain more than one meaningful concept, the number of codes exceeded the number of items.

### Data synthesis

After completing the linking process, a descriptive analysis was carried out to analyse the meaningful concepts and their links to the ICF. Concepts linked to each of the ICF components (b, d, s, e), chapters, and categories were summarised. Concepts classified as ‘not covered’ or ‘not definable’ were further categorised according to the ICF linking rules.

Due to the large number of items included, detailed analysis was limited to first- and second-level ICF categories while the total number of third-level categories for each component is reported. Percentage coverage of the ICF was calculated as the number of second-level categories linked to EORTC concepts out of the total number of second-level categories included in each ICF chapter and component. ICF categories ‘other specified’ and ‘unspecified’ were not included in this calculation. Frequency and distribution of coverage were presented by calculating the numbers of EORTC concepts linked to each ICF second-level category, domain, chapter, and component, in addition to percentages based on the total concepts linked to the ICF.

## Results

Agreement between the reviewers was 92.0% for level one categories, 88.6% for level two categories. Perfect agreement (100%) was achieved after consensus discussion.

Overall, 1860 concepts were extracted from a total of 1076 EORTC items (all EORTC Item Library content as of December 2025). Approximately half of the items (n = 534, 49.6%) included one concept, while 542 (50.4%) included more than one concept. The EORTC concepts were linked to most ICF chapters (28/30, 93.3%), except for chapters s1 Structures of the nervous system and s4 Structures of cardiovascular, immunological and respiratory systems. EORTC concepts were linked to a total of 168 (60.9%) second-level ICF categories, out of the 276 categories currently included within the ICF (Tables 2, 3, 4, 5). The component of ‘b—Body functions’ had the highest coverage, with concepts linked to 80.0% (64/80) of all second-level categories, followed by ‘d—Activities and participation’ with a total coverage of 68.5% (63/92). EORTC concepts were linked to all (100%) second-level categories in chapters: b6 Genitourinary and reproductive functions, b8 Functions of the skin and related structures, d2 General tasks and demands, d5 Self-care, and d6 Domestic life had perfect coverage. The component of ‘s-Body structures’ was the least covered with EORTC concepts linked to only 35.0% (14/40) of all second-level categories.

**Table 2:** EORTC content coverage of ICF component ‘b-Body functions’

Chapters	Coverage* n (%)	Frequency and distribution of linked concepts per chapter, domain, and category (% of total concepts linked to this component)				
		n (%)	Domains	n (%)	Level 2 categories	n (%)
b1 Mental functions	15 (83.3%)	249 (28.6%)	Global mental functions	89 (10.2%)	b110 Consciousness functions	4 (0.5%)
					b114 Orientation functions	4 (0.5%)
					b126 Temperament and personality functions	11 (1.3%)
					b130 Energy and drive functions	52 (6.0%)
					b134 Sleep functions	18 (2.1%)
			Specific mental functions	160 (18.3%)	b140 Attention functions	11 (1.3%)
					b144 Memory functions	23 (2.6%)
					b147 Psychomotor functions	3 (0.3%)
					b152 Emotional functions	77 (8.8%)
					b156 Perceptual functions	1 (0.1%)
					b160 Thought functions	6 (0.7%)
					b164 Higher-level cognitive functions	10 (1.1%)
					b167 Mental functions of language	5 (0.6%)
					b180 Experience of self and time functions	24 (2.8%)
b2 Sensory functions and pain	11 (91.7%)	171 (19.6%)	Seeing and related functions	36 (4.1%)	b210 Seeing functions	17 (2.0%)
					b215 Functions of structures adjoining the eye	6 (0.7%)
					b220 Sensations associated with the eye and adjoining structures	13 (1.5%)
			Hearing and vestibular functions	4 (0.5%)	b230 Hearing functions	1 (0.1%)
					b235 Vestibular Functions	1 (0.1%)
					b240 Sensations associated with hearing and vestibular function	2 (0.2%)
			Additional sensory functions	27 (3.1%)	b250 Taste function	3 (0.3%)
					b255 Smell function	4 (0.5%)
					b265 Touch function	3 (0.3%)
					b270 Sensory functions related to temperature and other stimuli	16 (1.8%)
					b279 Additional sensory functions, other specified and unspecified	1 (0.1%)
			Pain	104 (11.9%)	b280 Sensation of pain	89 (10.2%)
					b289 Sensation of pain, other specified and unspecified	15 (1.7%)
b3 Voice and speech functions	2 (50.0%)	5 (0.6%)	–	–	b310 Voice functions	4 (0.5%)
					b320 Articulation functions	1 (0.1%)
b4 Functions of the cardiovascular, haematological, immunological and respiratory systems	8 (80.0%)	109 (12.5%)	Functions of the cardiovascular system	6 (0.7%)	b410 Heart functions	4 (0.5%)
					b415 Blood vessel functions	2 (0.2%)
			Functions of the haematological and immunological systems	10 (1.1%)	b430 Haematological system functions	1 (0.1%)
					b435 Immunological system functions	9 (1.1%)
			Functions of the respiratory system	28 (3.2%)	b440 Respiration functions	28 (3.2%)
			Additional functions and sensations of the cardiovascular and respiratory systems	65 (7.5%)	b450 Additional functions of the respiratory system	10 (1.1%)
					b460 Sensations associated with cardiovascular and respiratory functions	31 (3.6%)
					b455 Exercise tolerance functions	24 (2.8%)
b5 Functions of the digestive, metabolic and endocrine system	7 (70.0%)	167 (19.2%)	Functions related to the digestive system	163 (18.7%)	b510 Ingestion functions	40 (4.7%)
					b515 Digestive functions	6 (0.7%)
					b525 Defecation functions	69 (8.1%)
					b530 Weight maintenance functions	15 (1.8%)
					b535 Sensations associated with the digestive system	31 (3.7%)
					b539 Functions related to the digestive system, other specified and unspecified	2 (0.2%)
			Functions related to metabolism and the endocrine system	4 (0.5%)	b540 General metabolic functions	1 (0.1%)
					b550 Thermoregulatory functions	3 (0.3%)
b6 Genitourinary and reproductive functions	7 (100.0%)	77 (8.8%)	Urinary functions	22 (2.5%)	b610 Urinary excretory functions	2 (0.2%)
					b620 Urination functions	18 (2.1%)
					b630 Sensations associated with urinary functions	2 (0.2%)
			Genital and reproductive functions	55 (6.3%)	b640 Sexual functions	34 (3.9%)
					b650 Menstruation functions	4 (0.5%)
					b660 Procreation functions	1 (0.1%)
					b670 Sensations associated with genital and reproductive functions	16 (1.8%)
b7 Neuromusculoskeletal and movement-related functions	8 (61.5%)	23 (2.6%)	Functions of the joints and bones	3 (0.3%)	b710 Mobility of joint functions	3 (0.3%)
			Muscle functions	10 (1.1%)	b730 Muscle power functions	7 (0.8%)
					b740 Muscle endurance functions	2 (0.2%)
					b749 Muscle functions, other specified and unspecified	1 (0.1%)
			Movement functions	10 (1.1%)	b760 Control of voluntary movement functions	3 (0.4%)
					b765 Involuntary movement functions	2 (0.2%)
					b770 Gait pattern functions	1 (0.1%)
					b780 Sensations related to muscles and movement functions	4 (0.5%)
b8 Functions of the skin and related structures	6 (100.0%)	71 (8.1%)	Functions of the skin	62 (7.1%)	b810 Protective functions of the skin	10 (1.1%)
					b820 Repair functions of the skin	13 (1.5%)
					b830 Other functions of the skin	5 (0.6%)
					b840 Sensation related to the skin	30 (3.4%)
					b849 Functions of the skin, other specified and unspecified	4 (0.5%)
			Functions of the hair and nails	9 (1.0%)	b850 Functions of hair	6 (0.7%)
					b860 Functions of nails	3 (0.3%)
Total coverage	64/80 (80.0%)				Total linked concepts	872

*Number and percentage of second-level categories covered per chapter (% = n covered categories/ n total ICF categories)

**Table 3:** EORTC content coverage of ICF component ‘d‐Activities and participation’

Chapters	Coverage* n (%)	Frequency and distribution of linked concepts per chapter, domain, and category (% of total concepts linked to this component)				
		n (%)	Domains	n (%)	Level 2 categories	n (%)
d1 Learning and applying knowledge	9 (42.9%)	16 (3.5%)	Purposeful sensory experiences	3 (0.7%)	d110 Watching	1 (0.2%)
					d115 Listening	1 (0.2%)
					d120 Other purposeful sensing	1 (0.2%)
			Basic learning	1(0.2%)	d155 Acquiring skills	1 (0.2%)
			Applying knowledge	12 (2.6%)	d160 Focusing attention	2 (0.4%)
					d166 Reading	4 (0.9%)
					d170 Writing	2 (0.4%)
					d175 Solving problems	2 (0.4%)
					d177 Making decisions	1 (0.2%)
					d199 Learning and applying knowledge, unspecified	1 (0.2%)
d2 General tasks and demands	4 (100.0%)	45 (9.9%)	–	–	d210 Undertaking a single task	3 (0.7%)
					d220 Undertaking multiple tasks	4 (0.9%)
					d230 Carrying out daily routine	33 (7.3%)
					d240 Handling stress and other psychological demands	5 (1.1%)
d3 Communication	9 (69.2%)	35 (7.5%)	Communicating – receiving	5 (1.3%)	310 Communicating with - receiving - spoken messages	1 (0.2%)
					d325 Communicating with - receiving - written messages	2 (0.4%)
					d329 Communicating with - receiving, other specified and unspecified	1 (0.2%)
			Communicating – producing	13 (2.9%)	d330 Speaking	10 (2.2%)
					d335 Producing nonverbal messages	1 (0.2%)
					d345 Writing messages	2 (0.4%)
			Conversation and use of communication devices and techniques	13 (2.9%)	d350 Conversation	10 (2.2%)
					d355 Discussion	3 (0.7%)
					d360 Using communication devices and techniques	3 (0.7%)
					d399 Communication, unspecified	2 (0.4%)
d4 Mobility	12 (75.0%)	105 (23.2%)	Changing and maintaining a body position	25 (5.5%)	d410 Changing basic body position	16 (3.5%)
					d415 Maintaining a body position	9 (2.0%)
			Carrying, moving and handling objects	21(4.6%)	d430 Lifting and carrying objects	12 (2.6%)
					d440 Fine hand use	3 (0.7%)
					d445 Hand and arm use	5 (1.1%)
					d446 Fine foot use	1 (0.2%)
			Moving around using transportation	7 (1.5%)	d470 Using transportation	1 (0.2%)
					d475 Driving	6 (1.3%)
			Walking and moving	49 (10.8%)	d450 Walking	23 (5.1%)
					d451 Going up and down stairs	13 (2.9%)
					d455 Moving around	5 (1.1%)
					d460 Moving around in different locations	8 (1.8%)
					d499 Mobility, unspecified	3 (0.7%)
d5 Self-care	7 (100.0%)	74 (16.3%)	–	–	d510 Washing oneself	5 (1.1%)
					d520 Caring for body parts	9 (2.0%)
					d530 Toileting	4 (0.9%)
					d540 Dressing	6 (1.3%)
					d550 Eating	27 (6.0%)
					d560 Drinking	5 (1.1%)
					d570 Looking after one's health	14 (3.1%)
					d599 Self-care, unspecified	4 (0.9%)
d6 Domestic life	6 (100.0%)	33 (7.3%)	Acquisition of necessities	6 (1.3%)	d610 Acquiring a place to live	2 (0.4%)
					d620 Acquisition of goods and services	4 (0.9%)
			Caring for household objects and assisting other	11(2.4%)	d650 Caring for household objects	5 (1.1%)
					d660 Assisting others	6 (1.3%)
			Household tasks	16 (3.5%)	d630 Preparing meals	2 (0.4%)
					d640 Doing housework	11 (2.4%)
					d649 Household tasks, other specified and unspecified	3 (0.7%)
d7 Interpersonal interactions and relationships	6 (85.7%)	88 (19.4%)	General interpersonal interactions	12 (2.6%)	d710 Basic interpersonal interactions	7 (1.5%)
					d720 Complex interpersonal interactions	3 (0.7%)
					d729 General interpersonal interactions, other specified and unspecified	2 (0.4%)
			Particular interpersonal relationships	75 (16.6%)	d740 Formal relationships	3 (0.7%)
					d750 Informal social relationships	22 (4.9%)
					d760 Family relationships	29 (6.4%)
					d770 Intimate relationships	17 (3.8%)
					d779 Particular interpersonal relationships, other specified and unspecified	1 (0.2%)
					d798 Other specified interpersonal interactions and relationships	1 (0.2%)
					d799 Interpersonal interactions and relationships, unspecified	3 (0.7%)
d8 Major life areas	7 (53.8%)	31 (6.8%)	Work and employment	16 (3.5%)	d845 Acquiring, keeping and terminating a job	6 (1.3%)
					d850 Remunerative employment	3 (0.7%)
					d855 Non-remunerative employment	1 (0.2%)
					d859 Work and employment, other specified and unspecified	6 (1.3%)
			Economic life	12 (2.6%)	d860 Basic economic transactions	3 (0.7%)
					d879 Economic life, other specified and unspecified	9 (2.0%)
			Education	3 (0.7%)	d839 Education, other specified and unspecified	3 (0.7%)
d9 Community, social and civic life	3 (60.0%)	35 (7.7%)	–	–	d910 Community life	11 (2.4%)
					d920 Recreation and leisure	32 (7.1%)
					d930 Religion and spirituality	3 (0.7%)
Total coverage	63/92 (68.5%)				Total linked concepts	453

*Number and percentage of second-level categories covered per chapter (% = n covered categories/ n total ICF categories)

**Table 4:** EORTC content coverage of ICF component ‘e-Environmental Factors’

Chapters	Coverage* n (%)	Frequency and distribution of linked concepts per chapter and category (% of total concepts linked to this component)		
		n (%)	Level 2 categories	n (%)
e1 Products and technology	7 (58.3%)	50 (23.0%)	e110 Products or substances for personal consumption	14 (6.5%)
			e115 Products and technology for personal use in daily living	22 (10.1%)
			e120 Products and technology for personal indoor and outdoor mobility and transportation	2 (0.9%)
			e125 Products and technology for communication	1 (0.5%)
			e130 Products for technology and education	1 (0.5%)
			e150 Design, construction and building products and technology of buildings for public use	2 (0.9%)
			e165 Assets	8 (3.7%)
e2 Natural environment and human-made changes to environment	3 (27.3%)	5 (2.3%)	e225 Climate	2 (0.9%)
			e240 Light intensity	2 (0.9%)
			e245 Time-related changes	2 (0.9%)
e3 Support and relationships	7 (63.6%)	96 (44.2%)	e310 Immediate family	3 (1.4%)
			e315 Extended family	1 (0.5%)
			e320 Friends	2 (0.9%)
			e325 Acquaintances, peers, colleagues, neighbours and community members	2 (0.9%)
			e340 Personal care providers and personal assistants	2 (0.9%)
			e355 Health professionals	80 (36.9%)
			e360 Other professionals	1 (0.5%)
			e398 Other specified support and relationships	2 (0.9%)
			e399 Support and relationships, unspecified	4 (1.9%)
e4 Attitudes	7 (58.3%)	9 (4.1%)	e450 Individual attitudes of health professionals	6 (2.8%)
			e455 Individual attitudes of other professionals	1 (0.5%)
			e499 Attitudes, unspecified	2 (0.9%)
e5 Service, systems and policies	3 (16.7%)	57 (26.3%)	e535 Communication services, systems and policies	1 (0.5%)
			e565 Economic services, systems and policies	2 (0.9%)
			e580 Health services, systems and policies	54 (24.9%)
Total coverage	27/64 (42.2%)		Total linked concepts	217

*Number and percentage of second-level categories covered per chapter (% = n covered categories/ n total ICF categories)

**Table 5:** EORTC content coverage of ICF component ‘s-Body structures’

Chapters	Coverage* n (%)	Frequency and distribution of linked concepts per chapter and category (% of total concepts linked to this component)		
		n (%)	Level 2 categories	n (%)
s1 Structures of the nervous system	0 (0.0%)		–	–
s2 The eye, the ear, and related structures	2 (33.3%)	9 (9.1%)	s220 Structure of eyeball	8 (8.1%)
			s230 Structures around eye	1 (1.0%)
s3 Structures involved in voice and speech	2 (50.0%)	14 (14.1%)	s310 Structure of nose	2 (2.0%)
			s320 Structure of mouth	12 (12.1%)
s4 Structures of cardiovascular, immunological and respiratory systems	0 (0.0%)		–	–
s5 Structures related to the digestive, metabolic and endocrine systems	1 (12.5%)	1 (1.0%)	s599 Structures related to the digestive, metabolic and endocrine systems, unspecified	1 (1.0%)
s6 Structures related to the genitourinary and reproductives systems	2 (66.7%)	29 (29.3%)	s620 Structure of pelvic floor	1 (1.0%)
			s630 Structure of reproductive system	28 (28.3%)
s7 Structures related to movement	4 (57.1%)	23 (23.2%)	s710 Structure of head and neck region	1 (1.0%)
			s730 Structure of upper extremity	7 (7.1%)
			s750 Structure of lower extremity	12 (12.1%)
			s770 Additional musculoskeletal structures related to movement	2 (2.0%)
			s798 Other specified structures related to movement	1 (1.0%)
s8 Skin and related structures	3 (75.0%)	23 (23.2%)	s810 Structure of areas of skin	13 (13.1%)
			s830 Structure of nails	4 (4.0%)
			s840 Structure of hair	6 (6.1%)
Total coverage	14/40 (35.0%)		Total linked concepts	99

*Number and percentage of second-level categories covered per chapter (% = n covered categories/ n total ICF categories)

Most concepts (1641/1860, 88.2%) were linked to an ICF second-level category (Tables 2, 3, 4, 5), while 20 represented ‘personal factors’ and 199 were not linked to an ICF category. Frequency of coverage showed that the ICF component with the most linked content was ‘b-Body functions’ (n = 872/1641, 53.1% of all concepts linked to ICF). This was followed by 453 (27.6%) concepts linked to ‘d—Activities and participation’, 217 (13.2%) linked to ‘e-Environmental factors’, and 99 (6.0%) concepts linked to ‘s-Body structures’. Of these, a total of 1149 concepts (70.0%) were linked to third-level ICF categories (n = 632 b codes, n = 327 d codes, n = 108 e codes, and n = 82 s codes).

Twenty EORTC items (1.9% of all items) included 20 concepts classified as ‘personal factors’ representing personal factors which may affect attitudes towards health, including motivation for treatment and religious and spiritual beliefs (Table 1).

The rest of the EORTC Item Library content (199/1860 concepts, 10.7%) was not linked to the ICF. Of these concepts, 152 (76.4%) were classified as ‘not covered’ because they were not covered in the ICF including general quality of life, health conditions, and satisfaction with care. Some of the concepts not covered in the ICF related to general treatment, including surgery and surgical procedures. A total of 47 concepts (23.6%) were classified as ‘not definable’ as they were too general to be linked to a specific ICF category. These concepts covered general health, physical health, and general symptoms or side effects. It should be noted that some of the items included concepts that were linked to one ICF category while also containing a concept that is not definable or not covered within the ICF.

## Discussion

This study systematically linked the EORTC Item Library to the ICF using the most recent ICF linking rules [19]. The results indicate that the Item Library provides broad coverage of the ICF, which reflects its inclusion of functioning, disability and health-related concepts associated with cancer and its treatment [28, 29]. This analysis provides support for the Item Library and its potential to be used as part of standardised health outcome reporting. Linking the EORTC Item Library with the ICF supports the conceptual clarity, comparability, and clinical relevance of EORTC-PROMs in oncology. The results may also improve uptake of EORTC-PROMs in oncology settings where the ICF is the main patient management framework, like rehabilitation services [18, 31].

The findings indicate that the EORTC Item Library demonstrates broad, though uneven, coverage of ICF concepts, reflecting its primary purpose as a patient-reported measure of HRQoL in oncology. Strong representation was observed for ICF components closely aligned with patients’ symptom experience and day-to-day functioning, mainly included within the ‘b-Body functions’ and ‘d‐Activities and participation’. Areas such as emotional functioning, fatigue, pain, mobility, self-care, and interpersonal relationships were frequently covered, underscoring the Item Library’s focus on outcomes that are directly meaningful to patients undergoing cancer and its treatment. The EORTC Item Library content was linked to all chapters in ‘b-Body functions’ which reflects wide coverage of impairment in various physiological functions of the human body. In contrast, there was limited coverage of ‘s-Body structures’, which is expected given that this component is focused more on specific anatomical classifications which are less relevant for EORTC measures where the intention is to capture individuals’ perceptions of their own health status and HRQoL. The relatively limited coverage of ‘e-Environmental factors’ is also not surprising given that EORTC measures are generally aimed at capturing aspects of health that change over time, supporting their use as outcome measures in clinical research and clinical practice, factors that tend to be more static are therefore not typically measured.

The distribution of coverage across ICF components also highlights important conceptual differences between the EORTC measures and the ICF framework. The ICF is a comprehensive classification system designed to describe functioning, disability, and health across biological, individual, and societal levels, whereas the EORTC Item Library is a measurement system intended to capture patients’ subjective perceptions of health status and HRQoL. This distinction is evident in the comparatively limited coverage of the ‘e-Environmental factors’, which includes chapters and categories related to services, policies, and broader contextual determinants of disability. While aspects of social support and healthcare professionals were commonly captured, wider environmental influences on functioning, which are central to the ICF, fall largely outside the scope of HRQoL assessment and are therefore less emphasised within the EORTC Item Library.

Overall, these results illustrate that the EORTC Item Library and the ICF are complementary rather than similar tools. The EORTC measures prioritise concepts most relevant to patients’ lived experiences of cancer, including symptoms, functional limitations, and psychosocial well-being, whereas the ICF offers a more neutral and exhaustive framework for describing functioning and disability across health conditions. Hence, this analysis identified many concepts covered in the EORTC items that are outside the scope of the ICF’s coverage, like general quality of life and satisfaction with care. Despite these differences in terms of scope, linking the two frameworks enhances conceptual clarity and supports the interpretability and comparability of EORTC outcomes, particularly in interdisciplinary and rehabilitation-oriented settings where the ICF is used for clinical reasoning and service planning.

The Item Library encompasses content that corresponds to what the ICF describes as personal factors [15]. These refer to individual characteristics and life experiences that shape how people perceive, experience, and respond to health, functioning, and disability, for example, individual beliefs and values, coping styles, and existential concerns such as religious or spiritual beliefs. The ICF does not formally classify personal factors primarily because of their extensive heterogeneity, strong cultural and contextual variability, and the lack of international consensus on their definition and categorisation [15, 32]. Moreover, formal classification of such factors raises methodological and ethical concerns, including the risk of normative judgment and reduced cross-cultural comparability. It has been widely argued that studies employing HRQoL as an endpoint should explicitly consider individuals’ religious, spiritual, and existential concerns, as these dimensions play a meaningful role in shaping subjective evaluations of HRQoL [33]. In the context of serious illness, drawing on spiritual beliefs to sustain hope and a sense of meaning has been described as an adaptive coping strategy and has been associated with improved psychological adjustment to cancer. Consistent with this perspective, a substantial proportion of items from the EORTC Spiritual Well-Being questionnaire (QLQ-SWB32) were primarily classified as personal factors related to religion, spirituality, existential meaning, and life beliefs [34].

Some EORTC items contain concepts that are not covered or not definable in ICF terms. These concepts include overarching aspects like HRQoL, general health, or physical health, in addition to content related to the health condition (cancer), treatment provided, and satisfaction with care. This reflects the purpose of the EORTC measures to capture the impact of cancer on general HRQoL, which is outside the scope of the ICF as a classification of functioning, disability and health. For example, while most of the items in the Patient Satisfaction with Cancer Care-Core questionnaire (QLQ-PATSAT33) could be linked to the ICF categories of health services and health professionals, the concepts of satisfaction with specific aspects of care were not included in the ICF categories.

This work complements previous publications linking items from the EORTC Item Library to the ICF, which reported that most items in the EORTC QLQ-C30 Core questionnaire and the EORTC CAT Core could be linked to ICF categories [22–25]. Unlike previous studies that focused on certain areas like physical functioning [23], emotional functioning [24], role and social functioning [22], and fatigue [25], this work provides a comprehensive overview of all EORTC concepts included within the current version of the Item Library. Due to the large number of items covered in the current study, we limited the analysis to level one and level two ICF categories, in contrast to previous studies which included more detailed analysis of ICF level three categories [35].

This study complements previous work where the EORTC Item Library was mapped to the content of the CTCAE framework [27], showing that the Item Library covers a wide range of adverse events related to cancer and its treatment. Linking the EORTC items to both the CTCAE and ICF highlights different aspects of conceptual coverage and supports the flexibility of their use. The CTCAE framework is more specific to oncology and covers mainly adverse events at the level of different human body systems and organs [36]. Conversely, the ICF, which is grounded in the same foundation as the ICD, is used globally across health conditions and settings to capture holistic aspects of functioning and disability related to health conditions. Its use helps to standardise data collection and reporting, while supporting integrated care services such as rehabilitation and supportive care.

### Strengths and limitations

Applying refined linking rules [19] enhanced transparency and reproducibility in our classification process. The linking rules have been widely used in research to link and analyse the content of hundreds of outcome measurement tools and clinical assessments and interventions across various health areas [37]. In this study, a large team of reviewers was utilised to ensure that each item was dually reviewed, and several meetings were carried out to achieve agreement of the linking results. Despite the methodological rigour of this method, subjective decisions remain probable; using a Delphi consensus technique in the future could improve robustness of the linking process. Indeed, this approach was successfully integrated in a separate study aimed at improving agreement on the coding of complex instruments in the vocational rehabilitation setting [38].

The results from this analysis will be integrated into the EORTC Item Library platform (itemlibrary.eortc.org)[9] which will allow users to view items based on ICF components, chapters, and categories. This classification will support the identification of items based on ICF codes, which may subsequently facilitate the creation and use of item lists tailored to users’ needs. As the main classification system for functioning, disability and health, the ICF is not intended to be used as a benchmark for HRQoL. Therefore, the observed gaps in coverage should be understood as reflecting intentional differences in scope rather than shortcomings of the EORTC Item Library as a measure of HRQoL. While we mapped concepts, we did not evaluate detailed gaps in coverage, as noted in previous literature [22, 23]. As such, the results of this study are not intended to be used to prioritise the development of new items to account for identified gaps in ICF coverage, however, users interacting with the interface may identify areas they deem relevant for future item development, which may expand upon the current ICF coverage. It should be noted that this analysis focused solely on item content, not considering the categorisation of response options which may be valuable for clinicians and researchers who need to identify specific items or instruments for a given purpose [37].

## Conclusions

Linking the EORTC Item Library to the ICF confirms and extends findings from prior research carried out as part of EORTC-PROM development and validation. The EORTC items and measures capture a broad array of concepts included within the ICF, reflecting a diverse representation of biopsychosocial aspects of functioning in persons with cancer. Moreover, the use of a standardised, international framework like the ICF will facilitate the selection of EORTC items and measures based on ICF categories, while providing a common language to support comparison with other instruments. This work supports the use of the EORTC Item Library for comprehensive PRO measurement in cancer research and care.

## Data Availability

The EORTC measures that support the findings of this study are available upon request from the EORTC Item Library website: https://itemlibrary.eortc.org/. All EORTC Quality of Life Group questionnaires are protected by copyright. All rights are reserved by the EORTC Quality of Life Group. No part of these instruments may be reproduced, translated, adapted, distributed, or used in any form without prior written permission from the EORTC Quality of Life Group. The EORTC Quality of Life Group’s business model involves charges for commercial companies using EORTC instruments. Academic use of EORTC instruments is free of charge.
